# Deletion of the NMDA Receptor GluN2A Subunit Significantly Decreases Dendritic Growth in Maturing Dentate Granule Neurons

**DOI:** 10.1371/journal.pone.0103155

**Published:** 2014-08-01

**Authors:** Timal S. Kannangara, Crystal A. Bostrom, Andrea Ratzlaff, Lee Thompson, Robyn M. Cater, Joana Gil-Mohapel, Brian R. Christie

**Affiliations:** 1 Division of Medical Sciences, University of Victoria, Victoria, Canada; 2 Department of Biology, University of Victoria, Victoria, Canada; 3 Neuroscience Graduate Program, University of Victoria, Victoria, Canada; 4 Department of Cellular and Physiological Sciences, University of British Columbia, Victoria, Canada; 5 Graduate Program of Neuroscience and The Brain Research Centre, University of British Columbia, Victoria, Canada; Institute for Interdisciplinary Neuroscience, France

## Abstract

It is known that NMDA receptors can modulate adult hippocampal neurogenesis, but the contribution of specific regulatory GluN2 subunits has been difficult to determine. Here we demonstrate that mice lacking GluN2A (formerly NR2A) do not show altered cell proliferation or neuronal differentiation, but present significant changes in neuronal morphology in dentate granule cells. Specifically, GluN2A deletion significantly decreased total dendritic length and dendritic complexity in DG neurons located in the inner granular zone. Furthermore, the absence of GluN2A also resulted in a localized increase in spine density in the middle molecular layer, a region innervated by the medial perforant path. Interestingly, alterations in dendritic morphology and spine density were never seen in dentate granule cells located in the outer granular zone, a region that has been hypothesized to contain older, more mature, neurons. These results indicate that although the GluN2A subunit is not critical for the cell proliferation and differentiation stages of the neurogenic process, it does appear to play a role in establishing synaptic and dendritic morphology in maturing dentate granule cells localized in the inner granular zone.

## Introduction

The generation of new neurons in the dentate gyrus (DG) of the hippocampus has been shown to persist throughout adulthood in many species [1]–[3], including humans [4]. These cells are believed to originate from radial glial-like neural stem cells located within the subgranular zone, where they proliferate, adopt a neuronal phenotype, and migrate into the granule cell layer (GCL) [5]–[7]. Immature neurons then proceed to sprout one or two primary dendrites that extend into the molecular layer, before maturing into neurons that are functionally and structurally indistinguishable from older neurons [8]–[10]. This process of structural plasticity, formally referred to as adult neurogenesis, is tightly regulated, and can be modulated by synaptic activity [11].

*N*-methyl-d-aspartate (NMDA) receptors, primarily known for their involvement in synaptic plasticity [12], [13] as well as learning and memory [14], have been also shown to modulate adult neurogenesis [15]–[19]. However, their exact role during this process is still under debate. For instance, under normal conditions, blocking NMDA receptor activity increases proliferation in the adult DG [15], [20]–[22] while in models of stroke and epilepsy, blocking these receptors inhibits the well known ischemic- and seizure-induced increase in cell proliferation [23], [24]. NMDA receptors have also been shown to play a role in dendritic morphology. For example, activation of NMDA receptors was shown to promote dendritic spine development [25], while inhibition of these ionotropic glutamate receptors results in increased spine stability in denervated mouse dentate granule cells (DGCs) and accelerated spine density recovery following entorhinal denervation [26]. Additionally, NMDA receptors are present in young DGCs even before spine formation, and the expression level of these receptors in young DGCs appears to be similar to that of mature DGCs [27]. Furthermore, while immature DGCs show drastic morphological changes after learning-induced activation of NMDA receptors, no such alterations are seen in mature cells following learning [28].

The NMDA receptor itself is a heterodimer, comprised of two obligatory GluN1 (previously NR1) subunits, and two regulatory subunits from the GluN2 family (GluN2A-D; formerly NR2A-D) or GluN3 family (GluN3A-B; formerly NR3A-B) [29]. The regulatory subunits impart distinctive functional changes on the NMDA receptor, which include altering the receptor's biophysical properties and affinity to intracellular binding proteins [29]. This certainly holds true for two of the most highly expressed regulatory subunits in the hippocampus, the GluN2A and GluN2B subunits [30].

The GluN2 subunits may be the key to understanding the different roles of NMDA receptors in adult neurogenesis. In addition to bestowing the conductance properties for NMDA receptors, they are also known to have a developmentally regulated expression pattern [31], [32]. During early neuronal development, the GluN2B subunit is predominantly expressed, while the GluN2A subunit has only minimal expression [31]. Later in development, GluN2A expression increases significantly until it is approximately equal to that of the GluN2B subunit [31]. While it is believed that this developmental pattern of expression of the GluN2B subunit is conserved during adult neurogenesis [33], the expression timeline of the GluN2A subunit in adult-born hippocampal neurons is currently unknown. If GluN2A expression increases late during adult neurogenesis, GluN2A-containing receptors may not play a prominent role during the early stages of neurogenesis [34]. However, the temporal characteristics of GluN2A subunit expression suggest that they may play an active role in the morphological maturation of neurons. Indeed, various studies have shown that the GluN2A subunit is critical for dendritic arbourization and spine formation [35]–[38].

In the present study, we show that the GluN2A subunit does not play a significant role in adult hippocampal neurogenesis. However, GluN2A deletion did produce significant alterations in dendritic morphology and spine density. Importantly, these deficits were restricted to a subpopulation of DGCs typically characterized by the presence of a single apical dendrite and localized within the inner granular zone (IGZ) of the DG. These results suggest that GluN2A-containing NMDA receptors play an important role during neuronal morphological maturation in the adult DG.

## Experimental Procedures

### 2.1 Animals and Housing Conditions

Adult (2–2.9 months) male wild-type (WT) and GluN2A knockout (GluN2A^−/−^) mice with a C57BL/6J background [39], [40] were housed in standard cages (2–3 mice per cage, minimal enrichment) in a colony maintained at 21°C. Animals were kept on a 12-hour light/dark cycle with access to food and water *ad libitum*. All procedures were approved by the University of Victoria Animal Care Committee [Protocol Number: 2013-001(1)] and were done in accordance with the Canadian Council on Animal Care. All efforts were made to minimize animal suffering and the number of animals used in these experiments.

### 2.2 Immunohistochemistry

#### 2.2.1 Tissue Preparation

GluN2A^−/−^ (n = 7) and WT littermate mice (n = 5) were briefly anaesthetized with isoflurane (Abbott Laboratories, North Chicago, IL, USA) and then received an intraperitoneal (i.p.) injection of urethane (250 mg/ml in water; 10 mg/kg of body weight). When deeply anaesthetized, mice were transcardially perfused with 0.9% NaCl followed by 4% paraformaldehyde (PFA). The brains were removed and left in 4% PFA overnight at 4°C and subsequently transferred to 30% sucrose. Following saturation in sucrose, serial coronal sections were obtained on a vibratome (Leica VT1000S, Nussloch, Germany) at a 30 µm thickness. Sections were collected into a 1/6 section-sampling fraction and stored in an anti-freeze cryoprotectant solution [0.04 M Tris-buffered saline (TBS), 30% ethylene glycerol, 30% glycerol] at 4°C.

#### 2.2.2 Immunohistochemistry

One in six sections were processed for detection of one of the following endogenous markers: 1) Ki-67, a nuclear protein expressed during all active phases of the cell cycle, but absent from cells at rest ([41], [42] for review, see [43]), 2) proliferating cell nuclear antigen (PCNA), which is expressed during all active phases of the cell cycle and for a short period of time after cells become post-mitotic (for review, see [43], [44]), and 3) neurogenic differentiation protein (NeuroD), a basic helix-loop-helix transcription factor involved in neuronal differentiation [45], [46].

Briefly, after thorough rinsing, sections that were processed for Ki-67 and PCNA were incubated twice in 10 mM sodium citrate buffer (in 0.1 M TBS, pH = 6.0) at 95°C for 5 minutes. For all immunohistochemical procedures, sections were then quenched with 3% H_2_0_2_/10% methanol in 0.1 M TBS for 15 minutes and pre-incubated for 1 hour with either 5% normal goat serum (for Ki-67 and PCNA) or 5% normal horse serum (for NeuroD). Sections were then incubated for 48 hours at 4°C with the respective primary antibody: 1) rabbit polyclonal anti-Ki-67 (1∶500; Vector Laboratories, Burlingame, CA, USA), 2) rabbit polyclonal anti-PCNA (1∶100; Santa Cruz Biotechnology, Santa Cruz, CA, USA), or 3) goat anti-NeuroD (1∶200; Santa Cruz Biotechnology). After thorough rinsing, sections were incubated for 2 hours with a biotin-conjugated secondary antibody: goat anti-rabbit IgG (1∶200; Vector Laboratories) for Ki-67 and PCNA, or horse anti-goat IgG (1∶200; Vector Laboratories) for NeuroD. Bound antibodies were visualized using an avidin-biotin-peroxidase complex system (Vectastain ABC Elite Kit, Vector Laboratories) with 2,2-diaminobenzidine (DAB; Vector Laboratories) as a chromogen. The sections were mounted onto 2% gelatin-coated microscope slides, dehydrated in a series of ethanol solutions of increasing concentrations followed by a 5 minutes incubation with a xylene substitute (CitriSolv, Fisher Scientific, Fair Lawn, NJ, USA), and coverslipped with Permount mounting medium (Fisher Scientific).

#### 2.2.3 Cell Quantification

All morphological analyses were performed on coded slides, with the experimenter blinded to animal identity, using an Olympus microscope (Olympus BX51, Center Valley, PA, USA) with 10x, 40x and 100x objectives. Image Pro-Plus software (version 5.0 for Windows TM, Media Cybermetic Inc., Silver Spring, MD, USA) and a Cool Snap HQ camera (Photometrics, Tucson, AZ, USA) were used for image capture. A modified stereological approach was used to estimate the total number of Ki-67-, PCNA-, and NeuroD-positive cells in the subgranular zone of the DG of the hippocampus following a procedure previously described by us [47]–[50] and others [51]–[53]. All sections containing the DG and spanning the entire dorsal/ventral axis of the hippocampus (from 1.34 mm posterior to Bregma, when both inferior and superior blades of the DG are present, to 3.52 mm posterior to Bregma, just before both inferior and superior blades of the DG connect on both extremities [54]) were used for the analysis, resulting in 9–10 DG sections per animal. In each section, all cells positive for either Ki-67, PCNA, or NeuroD that were present within two to three nuclear diameters below the GCL were counted. The results were expressed as the total number of labeled cells in the DG sub-region of the hippocampus by multiplying the average number of labeled cells/DG section by the total number of 30 µm thick-sections that contain the DG (estimated as 73 sections). Images were processed with Adobe Photoshop 4.0 (Adobe Systems Mountain View, CA, USA). Only contrast enhancements and colour level adjustments were made; otherwise images were not digitally manipulated.

### 2.3 Golgi Staining

#### 2.3.1 Golgi Impregnation

GluN2A^−/−^ (n = 6) and WT littermate mice (n = 6) were briefly anaesthetized with isoflurane (Abbott Laboratories) followed by an i.p. injection of urethane (250 mg/ml in water; 10 mg/kg of body weight). Mice were then transcardially perfused with 0.9% saline. Brains were immediately removed and placed in vials containing 20 mL of modified Golgi-Cox solution and stored at room temperature, in the dark [55]. This solution was replaced with fresh Golgi-Cox solution after 24 hours and stored at room temperature in the dark for 14 days. The brains were then transferred to 30% sucrose solution and stored in the dark at room temperature for a maximum of 21 days.

#### 2.3.2 Slice preparation and processing

Coronal sections (200 µm) were generated throughout the length of the hippocampus using a vibratome (Leica 1400). Sections were immediately mounted onto 2% gelatin-coated microscope slides and processed as previously described [56]. Briefly, sections were sequentially placed in: dH_2_O (1 min), ammonium hydroxide (30 min), dH_2_O (1 min), Kodafix for film (30 min), dH_2_O (1 min), 50% ethanol (1 min), 70% ethanol (1 min), 95% ethanol (1 min), 100% ethanol (2×5 min), 100% ethanol/HemoDe/chloroform (1∶1∶1 for 10 min) and finally HemoDe (2×15 min). After processing, all slides were coverslipped, using Permount mounting medium (Fisher Scientific) and stored in the dark.

#### 2.3.3 Dendritic analysis

All morphological analyses were performed on coded slides, with the experimenter blinded to animal identity, using an Olympus CX21 light microscope with 10x and 40x objectives. Eight to eleven DGCs from each WT and GluN2A^−/−^ brain (n = 6 per genotype) were classified into one of two groups based on the location of the soma within the GCL: outer granular zone (OGZ) cells had somas located in the outer half of the GCL (adjacent to the molecular layer), and IGZ cells had somas located within the inner half of the GCL [adjacent to the subgranular zone (SGZ) and hilus], as previously described [56]. Cells with somas located directly at the halfway point of the GCL (between the molecular layer and the hilus) were excluded. For this study, 4–6 OGZ and 4–5 IGZ cells were analyzed per animal. Cells were uniformly distributed through the dorsal-ventral axis of the DG. Golgi-impregnated cells were selected if they fulfilled the following criteria: 1) had consistent impregnation throughout the extent of the cell body and dendrites, 2) were distinguishable from neighboring impregnated cells, and 3) had intact dendritic trees. Each selected cell was traced by hand using a camera lucida projection from the microscope at 40x magnification. Cells were then scanned at 300 dpi and measured using the NeuroJ plugin [57] for ImageJ (Rasband W.S., ImageJ, NIH, Bethesda, Maryland, USA). Differences between WT and GluN2A^−/−^ cells were assessed by examining mean total dendritic branch length and branch order. A Sholl analysis [58] was performed by quantifying the number of dendritic processes crossing concentric circles located at 20 µm intervals. Digital images were obtained using a Cool Snap HQ CCD camera (Photometrics) and Image Pro-Plus software (version 5.0 for Windows TM, Media Cybermetic Inc.).

#### 2.3.4 Spine analysis

A subset of Golgi-impregnated cells was randomly chosen for spine density analysis using an Olympus BX51 microscope with 40x and 100x objectives, and a Retiga-2000R camera (QImaging, Surrey, BC, Canada). The DG molecular layer was subdivided into three equal regions. For this study, the most outer region (outer molecular layer), and the middle region (middle molecular layer) were investigated, as they are the location of the lateral perforant path, and medial perforant path, respectively. For each cell, three 10 µm dendritic segments were randomly chosen, under x100 magnification, from each region. Z-stacks (stack depth varied depending on dendrite segment) were obtained for each segment, and the number of spines per 10 µm was quantified using Neurolucida and NeuroExplorer morphometry software (MBF Bioscience, Williston, VT, USA).

### 2.4 Statistical Analysis

Computed results were processed for statistical analysis using Excel 2007 (Microsoft Office) and Statistica 7.0 (Statsoft Inc., Tulsa, OK, USA). For all studies, data were presented as mean ± standard error of the mean (S.E.M.). Individual unpaired, two-tail student's t-tests were performed for Ki-67, PCNA, NeuroD and dendritic branch length analyses. A repeated measures analysis of variance (ANOVA) was performed for Sholl analysis and branch order, using genotype and either distance from soma, or branch order, as factors for Sholl and branch order analysis, respectively. The repeated measures ANOVA was followed by planned comparisons of least square means between genotypes. A 2-tailed 3×2 Fisher's exact test was used to determine differences in the proportion of granule cells with different numbers of primary dendrites. Differences were considered to be statistically significant when *p*<0.05.

## Results

### 3.1 Cell proliferation and neurogenesis are intact in GluN2A^−/−^ mice

**Figure 1:**
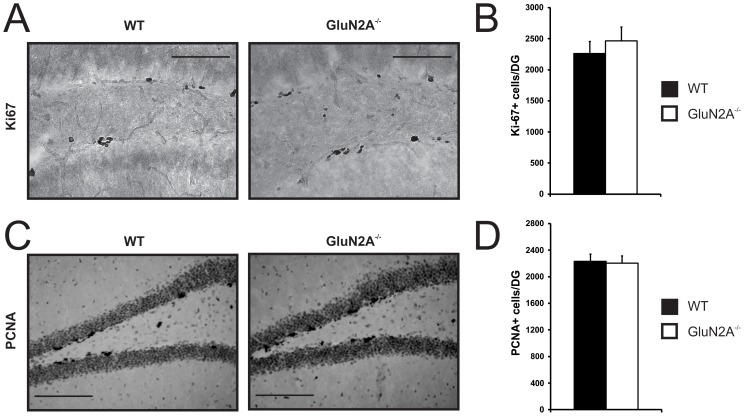
Intact rate of cell proliferation in the adult dentate gyrus of GluN2A^−/−^ mice. ***A, C,*** Representative images of dentate gyrus sections processed for Ki-67 (***A***) and PCNA (***C***) immunohistochemistry in both wild-type (WT, *left panels*) and GluN2A^−/−^ (*right panels*) mice. (***B, D***) No change in the number of Ki-67-positive cells (***B***) or PCNA-positive cells (***D***) in the adult dentate gyrus of GluN2A^−/−^ mice in comparison to wild-type littermates. For both graphs, WT: black; GluN2A^−/−^: white. Data is represented as means ± SEM. Scale bar  = 100 µm (***A, C***).

To assess whether the loss of the GluN2A subunit alters the levels of proliferation in the adult DG, we performed immunohistochemistry for the endogenous proliferation marker Ki-67, a nuclear protein expressed during all active phases of the cell cycle but absent in cells at rest [41], for review, see [43]. GluN2A^−/−^ mice showed a similar number of Ki-67-positive cells in comparison to their WT littermates (WT: 2263±193 Ki-67-positive cells, GluN2A^−/−^: 2464±225 Ki-67-positive cells, t_(9)_ = −0.683, *p*>0.5, **Figure 1A and B**). Immunohistochemistry for proliferating cell nuclear antigen (PCNA), another endogenous marker of cell proliferation that is expressed during all phases of the cell cycle [43]; for review see [42], confirmed these results (WT: 2230±114 PCNA-positive cells, GluN2A^−/−^: 2202±115 PCNA-positive cells, t_(9)_ = 0.169, *p*>0.5, **Figure 1C and D**). Together, these results strongly indicate that the absence of the GluN2A subunit does not alter cell proliferation in the adult DG.

**Figure 2:**
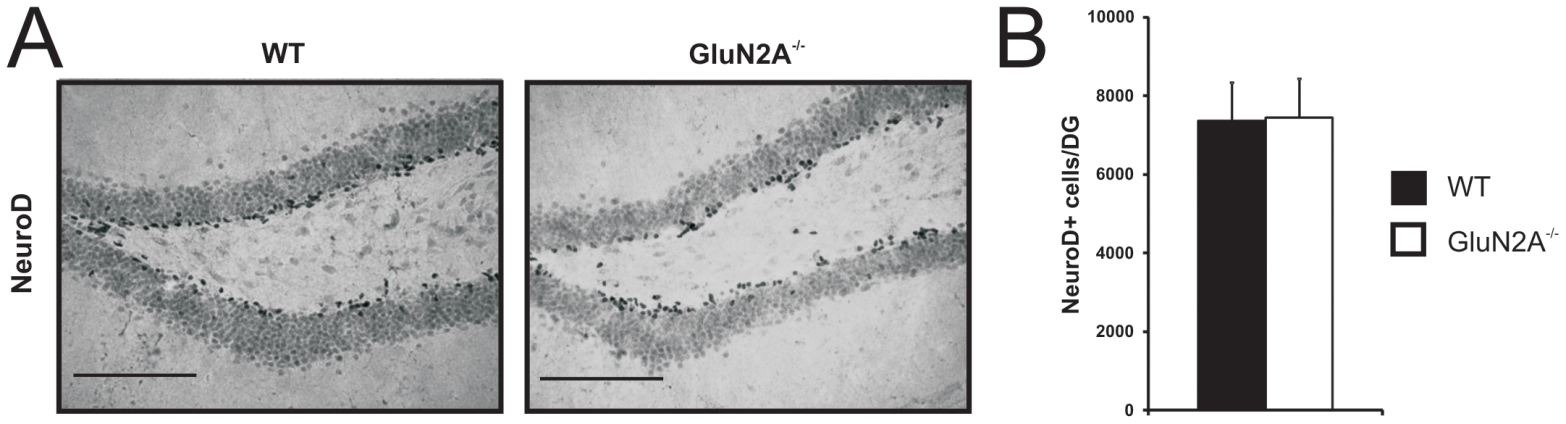
Intact rate of differentiation in the adult dentate gyrus of GluN2A^−/−^ mice. (***A***) Representative images of dentate gyrus sections processed for NeuroD immunohistochemistry in wild-type (WT, *left panel*) and GluN2A^−/−^ (*right panel*) mice. (***B***) No change in the number of NeuroD-positive cells in the adult dentate gyrus of GluN2A^−/−^ mice, in comparison to wild-type littermates. For graph, WT: black; GluN2A^−/−^: white. Data is represented as means ± SEM. Scale bar  = 100 µm (***A***).

The levels of neuronal differentiation were determined by examining the number of cells that express the immature neuronal marker NeuroD [45], [46]. GluN2A^−/−^ mice showed a similar number of NeuroD-positive cells in the SGZ of the hippocampal DG when compared with their WT littermate controls (WT: 7369±982 NeuroD-positive cells, GluN2A^−/−^: 7450±992 NeuroD-positive cells, t_(9)_ = −0.062, *p*>0.5, **Figure 2**). This result indicates that the number of differentiating neurons is also unchanged by GluN2A expression.

### 3.2 Impaired dendritic morphology in the inner layer of the DG granular zone of GluN2A^−/−^ mice

As no differences in the total numbers of proliferating cells and differentiating neurons were observed between WT and GluN2A^−/−^ mice, we next evaluated whether the absence of the GluN2A subunit had an effect in the morphology of DGCs. The DG follows an outside-in gradient with regards to cell age, with older cells located in the outer layer of the granular zone (i.e., in the OGZ), and younger cells located in the inner layer of the granular zone (i.e., in the IGZ) (for review, see [59]). Moreover, DGCs that are born during adulthood hardly ever reach the OGZ with the majority remaining within the inner third of the GCL (i.e., within the IGZ) by the time they finish their migration [60]. Thus, while the OGZ is composed by a more or less homogeneous group of DGCs that were generated during development, the IGZ is composed of a mixture of DGCs that were generated through both developmental and adult neurogenesis.

**Figure 3:**
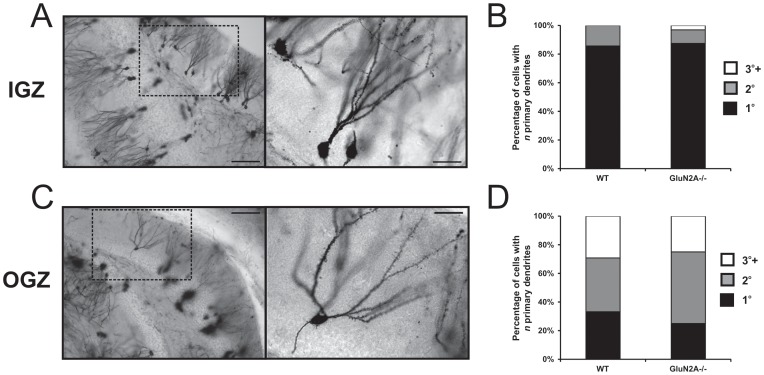
GluN2A does not alter the number of primary dendrites in dentate granule cells. (***A, B***) Representative images of Golgi-impregnated cells from the inner granular zone (IGZ, ***A***) and outer granular zone (OGZ, ***B***). Light micrographs were acquired at 10x (*left panels*) and 40x (*right panels*) magnification. (***C, D***) Percentage of dentate granule cells with different numbers of primary dendrites. GluN2A expression did not alter the number of cells with one (*black*), two (*grey*), and three or more (*white*) primary dendrites in IGZ (***C***) and OGZ (***D***) cells. Scale bar  = 100 µm (*left panels*), 25 µm (*right panels*) (***A, B***).

We used the Golgi impregnation technique to examine the number of primary dendrites present in DGCs that were located in the IGZ and the OGZ of both WT and GluN2A^−/−^ mice (**Figure 3A and B**). WT cells with somas located in the IGZ predominantly had one primary dendrite (1°: 85.7%, 2°: 14.3%. 3°+: 0.0%), while WT cells with somas located in the OGZ cells had multiple primary dendrites (1°: 33.3%, 2°: 37.5%, 3°+: 29.2%) (2-tailed 3×2 Fisher's exact test, *p*<0.001, **Figure 3C and D**). These results confirm the previously reported observation that younger DGCs (present in the IGZ and generated during both development and adulthood) usually present with few primary dendrites, whereas older DGCs (present in the OGZ and generated primarily during development) often have multiple primary dendrites [8], [56]. The percentage of DGCs with multiple primary dendrites was unchanged in GluN2A^−/−^ mice both in the IGZ (1°: 87.5%, 2°: 9.4%, 3°+: 3.1%; 2-tailed 3×2 Fisher's exact test, *p*>0.5, **Figure 3C**) and the OGZ (1°: 25%, 2°: 50%, 3°+: 25%; 2-tailed 3×2 Fisher's exact test, *p*>0.5, **Figure 3D**), indicating that the GluN2A subunit does not alter primary dendrite number.

**Figure 4:**
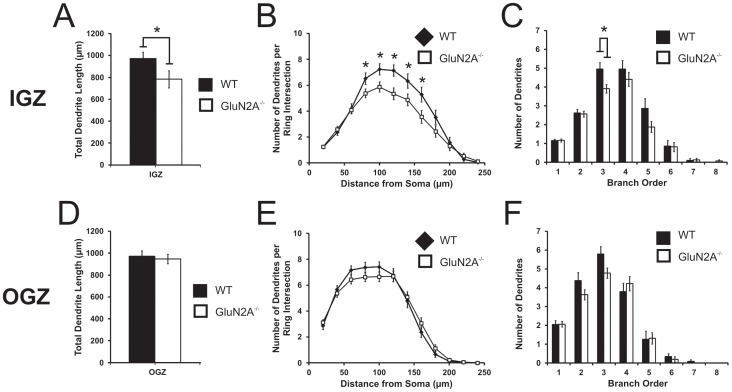
Altered dendritic morphology in IGZ dentate granule cells from GluN2A^−/−^ mice. (***A, D***) Total dendritic length was significantly decreased in GluN2A^−/−^ inner granular zone (IGZ) cells (***A***) and unaltered in GluN2A^−/−^ outer granular zone (OGZ) cells (***D***). ***B, E,*** Sholl analysis of IGZ (***B***) and OGZ (***E***) cells. A repeated measures ANOVA revealed a main effect of genotype in IGZ cells (***B***), with GluN2A^−/−^ IGZ cells having a lower number of dendrites per ring intersection from 80–160 µm from soma. No effect of genotype was seen in OGZ cells (***E***). (***C, F***) Branch order analysis of IGZ (***C***) and OGZ (***F***) cells. A repeated measures ANOVA revealed a main effect of genotype in IGZ cells (***C***), showing a significantly less number of third-order branches in GluN2A^−/−^ IGZ cells. OGZ cells did not show a main effect of genotype (***F***). For all graphs, WT: black; GluN2A^−/−^: white. Data is represented as means ± SEM. * denotes statistical significant difference (*p*<0.05).

To determine the contribution of GluN2A to dendritic morphology, we also examined the length and complexity of dendritic arbours. Interestingly, a significant decrease in total dendritic length was observed in DGCs located in the IGZ of GluN2A^−/−^ mice (WT: 972.41±57.39 µm, GluN2A^−/−^: 783.18±77.11 µm, t_(51)_ = 2.417, *p*<0.05, **Figure 4A**). A Sholl analysis revealed main effects of both genotype (F_(1, 51)_ = 5.26, *p*<0.05, **Figure 4B**;) and distance from soma (F_(11, 561)_ = 109.8, *p*<0.0001) in these younger cells. Further analysis revealed significant reductions in dendritic complexity between 80 to 160 µm from the soma in IGZ GluN2A^−/−^ cells (*p*<0.05 for each 80–160 µm comparison). Branch order analysis also revealed a main effect of genotype in IGZ cells (F_(1, 51)_ = 4.17, *p*<0.05, **Figure 4C**), with a significant reduction observed in third order branches in IGZ cells from GluN2A^−/−^ mice (F_(1, 51)_ = 7.25, *p*<0.05).

In contrast to IGZ cells, no differences in dendritic length were found between DGCs located in the OGZ of GluN2A^−/−^ mice and their WT counterparts (WT: 971.61±50.56 µm, GluN2A^−/−^: 946.56±42.56 µm, t_(54)_ = 0.397, *p*>0.5, **Figure 4D**). A Sholl analysis only revealed a main effect of distance from the soma (F_ (10, 530)_ = 192.68, *p*<0.0001, **Figure 4E**), with no significant reductions in dendritic complexity between genotypes. Similarly, branch order analysis did not reveal a main effect of genotype, only branch order (F_(6, 424)_ = 105.80, *p*<0.0001, **Figure 4F**).

### 3.3 Neurons located in the inner layer of the DG granular zone of GluN2A^−/−^ mice show an increase in spine density

**Figure 5:**
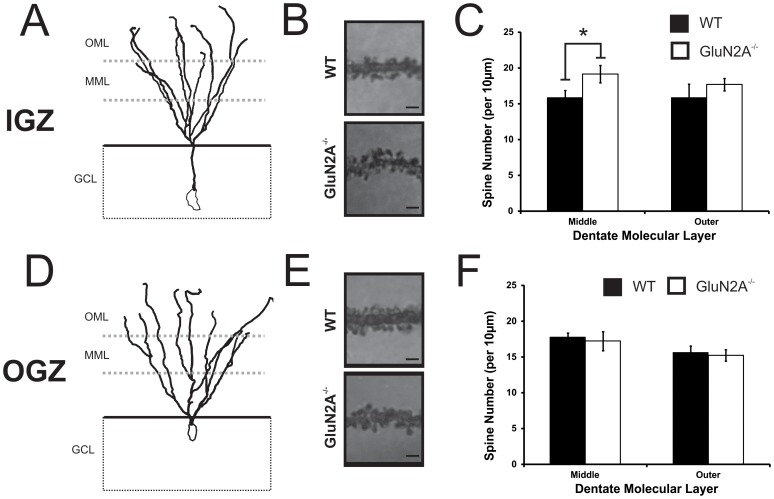
IGZ dentate granule cells show an increase in spine density in adult GluN2A^−/−^ mice. (***A, D***) Representative tracings of inner granular zone (IGZ, ***A***) and outer granular zone (OGZ, ***D***) cells produced using Neurolucida. Three dendritic regions were chosen from both the middle molecular layer (MML) and outer molecular layer (OML) of the dentate gyrus. (***B, E***) Representative images of dendritic regions from wild-type (WT, *upper panel*) and GluN2A^−/−^ (*lower panel*) granule cells located in the IGZ (***B***), and OGZ (***E***). (***C, F*** **)** Spine density of IGZ (***C***) and OGZ (***F***) cells. An increase in spine density was observed in IGZ cells from GluN2A^−/−^ mice, but only in the middle molecular layer (***C***). No changes in spine density were seen in OGZ cells, in either region of the molecular layer (***F***). GCL =  Granular Cell Layer. Data is represented as means ± SEM. Scale bar  = 1 µm (**B, E**). * denotes statistical significant difference (*p*<0.05).

DGCs project their dendrites into the full extent of the DG molecular layer. Therefore, dendritic spine density was analyzed in two locations of the molecular layer, the middle molecular layer and the outer molecular layer, where DGCs receive afferent input from the medial perforant path and the lateral perforant path, respectively. GluN2A^−/−^ cells with the soma located in the IGZ showed approximately a 21% increase in spine density in dendrites sampled from the middle molecular layer (WT: 15.8±1.00, GluN2A^−/−^: 19.1±1.22, t_(21)_ = −2.16, *p*<0.05, **Figure 5C**), but not from the outer molecular layer (WT: 15.8±1.93, GluN2A^−/−^: 17.7±0.88, t_(20)_ = −1.25, *p*>0.1). In comparison, GluN2A^−/−^ cells with the soma located in the OGZ showed no differences in spine density in dendrites sampled from either the middle molecular layer (WT: 17.8±0.59, GluN2A^−/−^: 17.0±0.90, t_(33)_ = 0.69, *p*>0.5, **Figure 5F**) or the outer molecular layer (WT: 15.6±0.92, GluN2A^−/−^: 15.2±0.80, t_(32)_ = 0.33, *p*>0.5).

## Discussion

The current results indicate that the GluN2A subunit of the NMDA receptor plays an important role in shaping the morphological characteristics of DGCs in the IGZ. Deletion of the GluN2A subunit did not significantly alter adult hippocampal neurogenesis (in particular the rates of cell proliferation and neuronal differentiation) Interestingly, the morphological effects of GluN2A deletion were restricted to a subpopulation of DGCs located within the IGZ. This subpopulation of DGCs is thought to represent a population of neurons that are primarily born later on and that include a significant proportion of newer neurons [8], [60], These results suggest that the GluN2A subunit plays a critical role in dendritic and spine development in maturing neurons.

### 4.1 Role of the GluN2A subunit during adult hippocampal neurogenesis

In this study, we did not observe any changes in the number of proliferating cells and immature neurons in GluN2A^−/−^ mice. This was apparent with both independent endogenous cell cycle markers (Ki-67 and PCNA; **Figure 1**) and the immature neuronal marker NeuroD (**Figure 2**). This is in agreement with the finding that the NMDA receptor and its major hippocampal subunits are not significantly expressed during the earliest stages of neurogenesis (i.e., in type-2a, type-2b and type 3 cells), although GluN1 and GluN2B subunits are minimally present in type-1 radial glia-like neural stem cells [33]. While it appears likely that GluN2A subunits do not play a role in adult neurogenesis, from these results we can not rule out the possibility that other subunits (*e.g.*, GluN2B) may be able to compensate for the absence of GluN2A.

Our findings support a previous study that also found unaltered cell proliferation in GluN2A^−/−^ mice using the exogenous mitotic marker 5-bromo-2′-deoxyuridine (BrdU) [34]. While the number of proliferating cells in our study is significantly higher than the one reported in that study [34], this discrepancy can be attributed to differences in the mitotic markers used. Both of the endogenous proliferation markers (i.e., Ki-67 and PCNA) used in the present study are expressed throughout all active phases of the cell cycle, while the exogenously administered BrdU (used in the study by Kitamura and colleagues; [34]) is only incorporated into the DNA of proliferating cells during S-phase. Thus BrdU only labels a subset of mitotic cells at any given time [43]. Nevertheless, in both studies no significant differences in the number of proliferating cells were observed between WT and GluN2A^−/−^ groups, regardless of the proliferation marker used. Furthermore, while others have suggested that blocking GluN2A-containing receptors may produce subtle decreases in cell proliferation in the DG [61], It is known that NVP-AAM077, the antagonist that was used to block GluN2A-containing receptors [48], has a low specificity for GluN2A subunits and can also block GluN2B subunits [62].

NMDA receptors are present in young (i.e., immature) DGCs before spine formation [27]. The expression of the immature neuronal marker NeuroD coincides with a significant increase in the expression of both GluN1 and GluN2B subunits [33]. While it is currently unclear when GluN2A expression begins in adult born DGCs, it has been hypothesized that the adult neurogenesis timeline of GluN2A expression may mirror other systems during development, where the GluN2A subunit only starts being strongly expressed once both GluN1 and GluN2B subunits have been expressed [63], [64]. Furthermore, GluN2A-containing receptors appear to be present only after synapse formation and not before (i.e., in mature neurons) [65], [66]. This would indicate that strong GluN2A expression occurs after neuronal commitment and therefore this subunit does not appear to be critical during this process. This is in agreement with our results demonstrating unchanged numbers of differentiating neurons in the GluN2A^−/−^ DG.

### 4.2 Role of the GluN2A subunit in the dendritic morphology of DGCs

Previous reports examining several neuronal populations [67], [68], including DGCs [28], [69], have suggested that NMDA receptor activity can modulate dendritic branching. Only recently have the roles of specific NMDA receptor subunits in neuronal morphology been investigated, and the findings are thus far unclear. Disrupting GluN2B expression alters dendritic arbourization in neurons from the *Xenopus* tectum [70], the trigeminal nucleus [71], and ventral spinal cord [72], but fails to produce significant changes in hippocampal cells [72], [73]. In the DG, GluN2B knockdown reduces the percentage of DGCs with multiple primary dendrites; interestingly, this effect was only observed in embryonic-born but not in adult-born DGCs [74].

Similarly, there is no consensus as to the role of GluN2A-containing receptors in dendritic morphology. Both overexpression and knock-down of GluN2A in *Xenopus* tectal neurons decreases branch clustering without modifying dendritic length [70]. Silencing GluN2A expression increases both dendritic length and complexity in hippocampal neural cultures [72], but does not alter dendritic structure in CA1 pyramidal cells [73]. Here we report that GluN2A deletion alters dendritic morphology in a specific population of DGCs located in the IGZ of the adult mouse hippocampal DG and thought to represent a younger population of maturing neurons [8], [60]. In GluN2A^−/−^ mice, these “younger” DGCs showed significant reductions in dendritic length and arbourization, which is in contrast to those located in the OGZ (and thought to constitute a more homogeneous population of “older” DGCs that were primarily generated during embryonic development). Overall, these studies suggest that the role of the GluN2A subunit in dendritic morphology may vary depending on the neuronal population [72]. Additionally, our finding alludes to a possible difference in the role of GluN2A on dendritic structure between the embryonic and adult neurogeneic processes, a difference also suggested previously with GluN2B [74]. In the adult DG, the GluN2A subunit appears to contribute to dendritic structure in a subset of “younger” DGCs located in the IGZ and possibly containing not only neurons that were generated during embryonic development but also those generated through the process of adult hippocampal neurogenesis. Future experiments are warranted in order to further elucidate the exact role of GluN2A in dendritic morphology.

### 4.3 Role of the GluN2A subunit in spinogenesis in DGCs

NMDA receptor activity is capable of modifying spine formation, as mice lacking cortical NMDA receptors have decreased spine density and altered spine morphology [75]. With regards to specific NMDA receptor subunits, it had been reported that GluN2B deletion decreases spine density [36], [73], [76], [77], however the role of GluN2A is less clear. The early induction of GluN2A expression in hippocampal neurons causes a significant decrease in spine density, while GluN2A knock-down produces a trend towards an increase in the number of new spines, suggesting that the GluN2A subunit may have an inhibitory role on spine density [36]. In contrast to this finding, silencing GluN2A expression does not alter spine density in CA1 pyramidal neurons [73].

Here, we detected a specific increase in spine density in GluN2A^−/−^ neurons that were located in the IGZ and projected their dendrites to the middle molecular layer. This increase in spine density further supports the idea that the presence of the GluN2A subunit may have an inhibitory effect on spine density in specific neuronal populations [35], [36]. While it is presently unclear why the loss of the GluN2A subunit specifically affected dendrites located within the middle molecular layer, this finding may be attributed to the type of afferent input that dendrites within this region receive. The DG receives major excitatory input from the perforant path, which can be subdivided into medial and lateral pathways based on anatomical and physiological properties. The medial perforant path innervates DGC dendrites in the middle molecular layer and demonstrates substantial NMDA receptor-dependent long-term potentiation, while the lateral perforant path innervates the outer molecular layer, and demonstrates less long-term potentiation, which can be regulated by opioid receptors [78], [79]. Thus, the number of NMDA receptors present at medial perforant path synapses in the middle molecular layer, may be higher than the number of NMDA receptors present at lateral perforant path synapses, in the outer molecular layer. In agreement, autoradiography experiments indicate a higher density of NMDA receptors in the inner half of the molecular layer in comparison to the outer half [80]. Thus, the higher concentration of NMDA receptors at spines in dendrites located in the middle molecular layer suggests that alterations in the expression of particular NMDA receptor subunits may have a greater impact on these spines.

These results further illustrate that GluN2A subunit appears to be of particular importance for the morphology of a subpopulation of “younger” DGCs, as these alterations in spine density were also restricted to DGCs with somas located within the IGZ and no differences in the number of spines was observed in DGCs with somas located within the OGZ.

### 4.4 Limitations and conclusions

The present study revealed deficits in the dendritic morphology of a subpopulation of DGCs specifically located in the IGZ of the mouse GluN2A^−/−^ hippocampus. Interestingly, these deficits were not observed in cells located in the OGZ, which is thought to be composed of a more or less homogeneous population of “older” DGCs that were generated during the period of brain development. While the reasons underlying the selective susceptibility of IGZ DGCs to the absence of the GluN2A subunit are currently unknown, it is possible that “older” cells (generated earlier during development and therefore localized in the OGZ) might have developed a mechanism to compensate for the lack of the GluN2A subunit (for example by primarily utilizing GluN2B-containing NMDA receptors with regards to the modulation of dendritic morphology). An alternative possibility is that the loss of GluN2A subunit may specifically affect the “younger” DGCs located in the IGZ by delaying their morphological (i.e., dendritic) maturation. This is a particularly interesting hypothesis given that the IGZ is formed by a more heterogeneous population of DGCs, some of which were generated during embryonic development (presumably during the later stages of development, given that the GCL is organized according to an outside-in gradient with regards to cell age, with older cells located in the OGZ, and younger cells located in the IGZ; for review, see [59]), and some having been generated during postnatal life, through the process of adult neurogenesis [60]. As such, it is tempting to speculate that the absence of the GluN2A subunit might be particularly detrimental to these “younger” cells, particularly those that were adult-born. Indeed, the appearance of multiple dendrites that extend the full extent of the molecular layer to the hippocampal fissure [81], the presence of spines in each dendrite [82], and the location of the somas within the IGZ [56] suggest that these IGZ neurons are less mature (and therefore “younger”) than those located in the OGZ. If this is indeed the case, then the absence of the GluN2A subunit may preferentially affect the morphological (i.e., dendritic) maturation of adult-born DGCs, which may be related with the fact that at this stage in neuronal maturation the expression of this subunit becomes more prominent (assuming that the expression pattern of the GluN2A subunit in adult-born neurons follows that of the developmentally generated ones [63], [64]).

However, it is important to keep in mind that the morphological characteristics and location within the GCL of a specific neuron do not allow us to determine its exact age (and stage of development) and more accurate techniques are required in order to exactly determine the age of the IGZ neurons that were examined in this study. The Golgi impregnation technique however, does not allow for the co-labeling of cells with a second neuronal marker (such as NeuroD or doublecortin for immature neurons and NeuN for mature neurons), and therefore it is impossible to determine the exact age of Golgi-impregnated neurons. Therefore, alternative techniques for labeling adult born granule neurons (e.g. retrovirus-mediated GFP expression and dye injections) followed by subsequent labeling with different differentiation and maturation markers (e.g., NeuroD, doublecortin, and NeuN) will be necessary to clarify whether the absence of the GluN2A subunit specifically affects the morphology of adult-born DGCs. Moreover, determining exactly when the expression of the GluN2A subunit is triggered in adult-born DGCs will also be important to further elucidate the role of this NMDA receptor subunit in the morphological maturation of these hippocampal neurons. Future studies are thus warranted in order to expand the results reported here. Nevertheless, the present findings indicate that the GluN2A subunit of the NMDA receptor plays a role in the dendritic morphology of a subset of “younger” DGCs that is specifically localized within the IGZ.

A second factor to be considered is that the global deletion of the GluN2A subunit may produce confounding effects on DGCs. For example, while gross brain anatomy is reported to be intact in GluN2A^−/−^ mice [40], afferent input into the hippocampus may be altered. It is unlikely that presynaptic changes are involved though, as presynaptic NMDA receptors in the perforant path-DGC synapse appear to only contain GluN2B subunits [83]. Future experiments using a mosaic model of GluN2A expression would aid in revealing whether the effects of the GluN2A subunit observed here are indeed cell autonomous.

In conclusion, we have shown that the levels of adult neurogenesis (in particular the rates of cell proliferation and neuronal differentiation) are unaltered in the hippocampal DG of GluN2A^−/−^ mice. However, absence of the GluN2A subunit altered specific morphological characteristics (namely dendritic length, branch arbourization, and spine density) in a subset of “younger” DGCs that is localized in the IGZ of the DG. These results point to a new role of GluN2A-containing NMDA receptors in morphological (i.e., dendritic) maturation.
